# Malignant Rhabdoid Tumor of the Lung in the Young Adult: A Case Report

**DOI:** 10.1155/2011/323584

**Published:** 2011-10-01

**Authors:** Adel Attia, Moosa Suleman, Hesham Mosleh

**Affiliations:** ^1^Pulmonology Department, King Fahad Specialist Hospital, Damman 31444, Saudi Arabia; ^2^Pathology Department, King Fahad Specialist Hospital, Damman 31444, Saudi Arabia

## Abstract

Malignant rhabdoid tumor (MRT) is one of the most aggressive and lethal malignancies in pediatric oncology. Malignant rhabdoid tumor was initially described in 1978 as a rhabdomyosarcomatoid variant of a Wilms tumor because of its occurrence in the kidney and because of the resemblance of its cells to rhabdomyoblasts. The absence of muscular differentiation led Haas and colleagues to coin the term rhabdoid tumor of the kidney in 1981, Haas et al..

## 1. Introduction

Rhabdoid tumor is a rare tumor that was first found to be in the kidney. Over the last several years, research has shown that it can also occur in the central nervous system (brain and spinal cord) as well as other locations outside the kidneys such as the liver, muscle, heart, lung, soft tissues, and skin. If the tumor is in the kidney, it is called “rhabdoid tumor of the kidney (RTK)”, and if it is in the brain or spinal cord, it is called “CNS atypical teratoid/rhabdoid tumors (AT/RTs)”. If it occurs outside of the kidney or brain, it has the longer name of ‘‘non-CNS extrarenal rhabdoid tumors” [1].

Considerable debate has been focused on whether extrarenal malignant rhabdoid tumors are the same as RTK. The recent recognition that CNS atypical teratoid/rhabdoid tumors (AT/RTs) have deletions of the *SMARCB1* gene indicates that rhabdoid tumors of the kidney and brain are identical or closely related entities. This observation is not surprising because rhabdoid tumors at both locations possess similar histologic, clinical, and demographic features. Moreover, 10%–15% of patients with malignant rhabdoid tumors have synchronous or metachronous brain tumors, many of which are second primary malignant rhabdoid tumors. Germline *SMARCB1* mutations were detected in approximately 15%–30% of these patients. *INI1* immunohistochemical studies can be used in conjunction with other studies to confirm the histologic diagnosis of malignant rhabdoid tumor [2].

## 2. Clinical History

**Figure 1:**
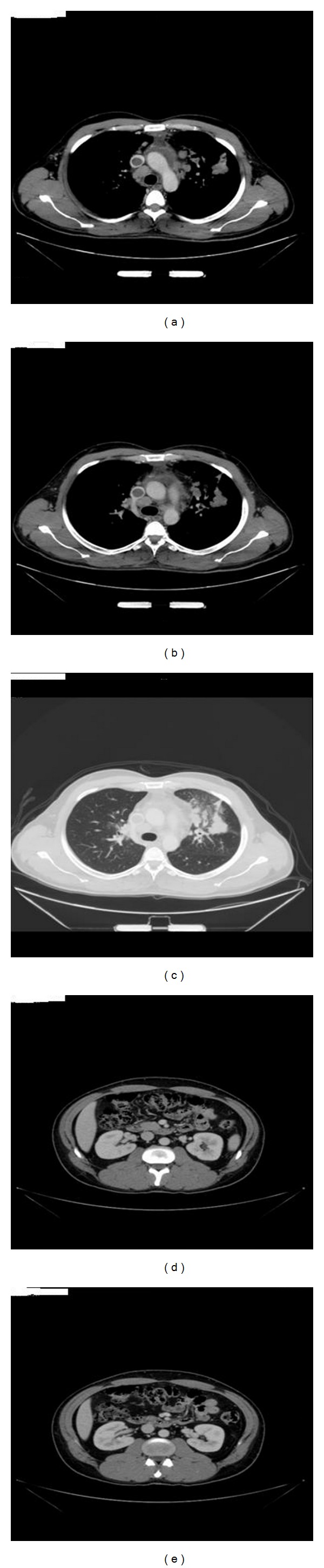
(a, b, and c) CT- chest showed left upper lobe mass mediastinal lymph nodes enlargement and superior vena cava thrombosis.(d and e) CT- abdomen showed spleen, pancreas, both adrenals, and both kidneys are normal. No ascites was noted.

This is a 36-year-old married male. He is nonsmoker and nondiabetic. The patient is not known to have any medical illness and was in his usual status till 6 months prior to the presentation, when he started to complain of subjective fever at night, night sweating, headache, and generalized weakness, but that did not interfere with his daily activity and neglect his condition until 2 months back, he started complaining of intermittent dull acting central chest pain that was not related to exertion and associated with mild shortness of breath and intermittent cough. He had lost about 10 kg in the last 2 months. There was no history of recent traveling or contact with febrile patient. No history of palpitations, apnea or PND. He sought advice in a private hospital, and they did chest X-ray and CT scan that showed left lung upper lobe mass, hilar and mediastinal lymphadenopathy, and superior vena cava thrombosis. The patient is on vitamin supplements and multiple antibiotics; he was given there. There was no past history of admission, surgical intervention, or blood transfusion (Figure 1).

## 3. Physical Examination

Temperature 37°C, pulse of 75 beats per minute, respiration 16 per minute, BP of 121/80 mmHg and oxygen saturation was 96% on room air. 

He is a young male, lying in bed, not in distress, conscious, alert, and oriented in time, place, and person.

Neck examination: Pemberton sign was positive.
Neurological examination: normal
Chest: showed normal S1 and S2, no murmur equal vesicular bilateral breath sounds with no adventitious sounds, and no conducting sounds.
Abdomen: soft, lax, no tenderness, and no organomegaly.

## 4. Investigations

CBC showed WBC of 6.45 × 10^9^/L, RBC 4.59 × 10^12^/L, hemoglobin of 11.9 g/dL, hematocrit 37.5%, MCV of 81.8 fL, MCH of 26 pcg, MCHC 31.8 g/dL, RDW 12.5%, platelet count of 453 × 10^9^/L, mean platelet volume of 8.4 fL, neutrophils of 60.8%, lymphocytes 18.7%, monocytes 10.4%, eosinophils 7.1%, basophils 1.7%, lymphocyte count absolute 1.21, monocytes 0.67, that is 670, eosinophils 0.46, basophils 0.11, atypical cells 1.2% and nucleated RBCs per high power field.

Liver panel: bilirubin of 6.3 mcmol/L, alkaline phosphatase 99 U/L, ALT of 24 U/L, AST of 12 U/L, GGT of 29 U/L, total protein of 79 g/L, albumin of 29 g/L, and albumin/globulin ratio was 0.6. 

Coagulation profile: PT of 13.45 sec, INR of 1.07 sec, and PTT of 36.41 sec. 

Renal panel: sodium of 137 mmol/L, potassium of 4.6 mmol/L, chloride of 102.1 mmol/L, bicarbonate of 27 mmol/L, BUN of 3.3 mmol/L, and creatinine of 86 mcmol/L. MDRD calculated was more than 60 ml/min. 

Bone panel: Phosphorus of 1.24 mmol/L, ALP of 101 U/L, total protein of 70 g/L, albumin of 31 g/L, calcium of 2.04 mmol/L and corrected calcium of 2.25. Magnesium of 0.95 mmol/L.

C-reactive protein = 80.9 mg/L. CMV IgG was reactive, CMV IgM non reactive. ESR of 16 mm/hr.

The chest X-ray revealed ill-defined area of consolidation seen at the left hilar region, no evidence of rib destruction, no pleural effusion in comparison to previous radiographs as per the radiologist. Abdominal ultrasound was done and did not show any abnormalities.

The patient underwent CT chest, abdomen, and pelvis. It showed left upper lung lobe mass, there was a nodular peribronchovascular infiltration involving the left lingular segment suggestive of lymphomatous infiltration, multiple enlarged mediastinal lymph nodes located at anterior mediastinal, paratracheal, peribronchial, subcarinal, and bilateral hilar groups, with the largest was at the paratracheal group measured 2.65 × 1.6 cm, still the thrombus in the right brachiocephalic vein and superior vena cava extended to the right atrium and the right subclavian vein with calcification of right axillary vein suggestive of chronic thrombosis with recanalization of the right subclavian vein. Minimal pericardial effusion was noted. No pleural effusion. The size and configuration of cardiac and mediastinal silhouette structures were maintained. The liver was normal in size in form of attenuation, no focal lesion, and no biliary dilatation. Inferior vena cava, hepatic, and portal vein were patent. Spleen, pancreas, both adrenals, and both kidneys were normal. No ascites was noted. The visualized thoracic bony cage, spine, and pelvis showed no focal bony lesions.

**Figure 2:**
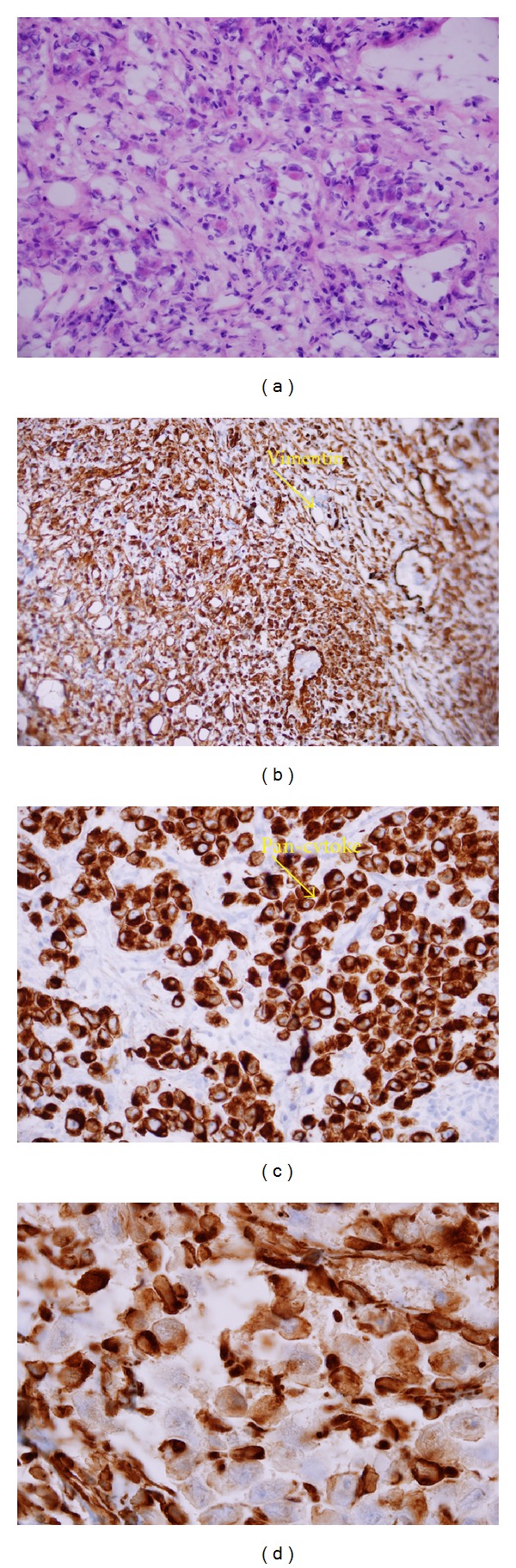
(a) Tumor cells with a cytoplasmic pink ball pushing the nucleus, (b) Pan Cytokeratin positivity, (c) Vimentin positivity, (d) Pan Cytokeratin high-power view.

The patient underwent bronchoscopy with transbronchial biopsy. The bronchoscopy showed the vocal cords were normal, trachea normal, carina shape normal, and in right and left lungs, no bronchial lesion was seen. Transbronchial biopsy was taken from the left upper lobe. The report of the bronchoalveolar lavage fluid showed excessive blood few scattered benign bronchial cells otherwise normal study. Microscopic description showed lung tissue with rhabdoid appearance, which was strongly positive for pan CK, vimentin, but negative for actin, myogenin, desmin, and myoglobin. Finally, the immunohistochemistry demonstrated lack of nuclear INI1 protein expression (Figure 2).

The patient underwent mediastinoscopy and the pathological report of the biopsy of the mediastinal lymph node that was taken on the mediastinoscopy. The biopsy specimen was reviewed, gross specimens consisted of mediastinal lymph nodes; measuring in aggregate 1.5 × 0.5 cm, frozen section diagnosis was metastatic tumor, whereas the specimen received in formalin consisted a piece of brownish tissue measuring 0.7 × 0.4 × 0.3 cm, grayish brown tissue measuring 0.9 × 0.8 × 0.3 cm. Microscopic description showed lymph nodes in all the above with metastasis with rhabdoid appearance, which was also strongly positive for pan CK, vimentin, but negative for actin, myogenin, desmin, and myoglobin. Finally, the immunohistochemistry demonstrated lack of nuclear INI1 protein expression.

Pathological diagnosis: Left lung upper lobe rhabdoid tumor with mediastinal lymph nodes nodal metastasis.

Diagnosis: left lung upper lobe rhabdoid tumor with multiple mediastinal lymphadenopathies with superior vena cava thrombosis.

## 5. Discussion

Malignant rhabdoid tumor was first identified in the kidney of infants and children and was described in 1978 as rhabdomyosarcomatoid variant of Wilms' tumor [3, 4]. However, because of the lack of ultrastructural or immunohistochemical evidence of myogenic differentiation, the term rhabdoid was later adapted for these neoplasms [5, 6]. With the same morphologic and immunohistochemical features, identical tumors have been reported in a wide variety of sites including the nervous system, eye, tongue, nasopharynx, neck, mediastinum, thymus, heart, uterus, urinary bladder, vulva, skin, soft tissue, paravertebral region, and gastrointestinal tract [4, 7]. Extrarenal rhabdoid tumours, although histologically, clinically, and ultrastructurally resembling renal rhabdoid tumours, are less consistent in presentation. Lung rhabdoid tumour is a rare histological finding especially in young adults. We did extensive research for malignant rhabdoid tumor of the lung, we did not find any publications for lung rhabdoid tumor.

The median age at presentation is 10.6 months, with a mean age of 15 months. Most patients are younger than 2 years. Malignant rhabdoid tumor has been reported in children older than this and in adults. 

As concern chemoradiotherapy, no consistently effective regimen has yet been established, and a standard regimen is not yet established.

 The prognosis for children with malignant rhabdoid tumors remains fair to poor, depending on the stage of the tumor at presentation, the patient's age at diagnosis, and possibly the genetic background.

## References

[1] Haas JE, Palmer NF, Weinberg AG, Beckwith JB (1981). Ultrastructure of malignant rhabdoid tumor of the kidney. A distinctive renal tumor of children. *Human Pathology*.

[2] Roberts CWM, Biegel JA (2009). The role of SMARCB1/INI1 in development of rhabdoid tumor. *Cancer Biology and Therapy*.

[3] Beckwith JB, Palmer NF (1978). Histopathology and prognosis of Wilms tumor. Results from the first national Wilms’ tumor study. *Cancer*.

[4] Yuri T, Danbara N, Shikata N (2004). Malignant rhabdoid tumor of the liver: case report and literature review. *Pathology International*.

[5] Amrikachi M, Ro JY, Ordonez NG, Ayala AG (2002). Adenocarcinomas of the gastrointestinal tract with prominent rhabdoid features. *Annals of Diagnostic Pathology*.

[6] Ogino S, Ro TY, Redline RW (2000). Malignant rhabdoid tumor: a phenotype? An entity? A controversy revisited. *Advances in Anatomic Pathology*.

[7] Chen Y, Jung SM, Chao TC (1998). Malignant rhabdoid tumor of the small intestine in an adult: a case report with immunohistochemical and ultrastructural findings. *Digestive Diseases and Sciences*.

